# Coadaptation Between Smart Technologies and Older Adults Over Time: Protocol for a Scoping Review

**DOI:** 10.2196/51129

**Published:** 2023-10-09

**Authors:** Kristina Marie Kokorelias, Alisa Grigorovich, Maurita T Harris, Umair Rehman, Louise Ritchie, AnneMarie Levy, Kerstin Denecke, Josephine McMurray

**Affiliations:** 1 Division of Geriatric Medicine Department of Medicine Sinai Health System and University Health Network Toronto, ON Canada; 2 Department of Occupational Science & Occupational Therapy Temerty Faculty of Medicine University of Toronto Toronto, ON Canada; 3 National Institute on Ageing Toronto Metropolitan University Toronto, ON Canada; 4 Toronto Rehabilitation Sciences Institute Temerty Faculty of Medicine University of Toronto Toronto, ON Canada; 5 KITE Research Institute Toronto Rehabilitation Institute University Health Network Toronto, ON Canada; 6 Recreation and Leisure Studies Brock University St. Catharines, ON Canada; 7 User Experience Design Wilfrid Laurier University Waterloo, ON Canada; 8 Department of Computer Science University of Western Ontario London, ON Canada; 9 Alzheimer Scotland Centre for Policy and Practice University of West Scotland Scotland United Kingdom; 10 Lazaridis School of Business & Economics/Community Health Wilfrid Laurier University Waterloo, ON Canada; 11 Institute for Medical Informatics Bern University of Applied Sciences Bern Switzerland

**Keywords:** scoping review, review methods, review methodology, knowledge synthesis, scoping, coadaptation, older adults, older adult, gerontechnology, technology, smart technology, smart technologies, smart, geriatrics, elderly, elder, geriatric, scoping literature review, protocol, internet of things, IoT, ageing, aging, PRISMA-ScR, user-centered design

## Abstract

**Background:**

The Internet of Things (IoT) has gained significant attention due to advancements in technology and has potential applications in meeting the needs of an aging population. Smart technologies, a subset of IoT, can support older adults in aging in place, promoting independent living and improving their quality of life. However, there is a lack of research on how older adults and smart technologies coadapt over time to maximize their benefits and sustain adoption.

**Objective:**

We will aim to comprehensively review and analyze the existing scientific literature pertaining to the coadaptation between smart technologies and older adults. The primary focus will be to investigate the extent and nature of this coadaptation process and explore how older adults and technology coevolve over time to enhance older adults' experience with technology.

**Methods:**

This scoping review will follow the methodology outlined in the Joanna Briggs Institute Reviewer's Manual and adhere to the PRISMA-ScR (Preferred Reporting Items for Systematic Reviews and Meta-Analysis Extension for Scoping Reviews) guidelines for reporting. Peer-reviewed articles will be searched in databases like Ovid MEDLINE, OVID Embase, PEDro, OVID PsycINFO, EBSCO CINAHL, the Cochrane Library, Scopus, IEEE Xplore, Web of Science, and Global Index Medicus. The research team will create a data extraction form covering study characteristics, participant characteristics, underlying models and frameworks, research findings, implications for technology coadaptation, and any identified study limitations. A directed content analysis approach will be used, incorporating the Selection, Optimization, and Compensation framework and Sex- and Gender-Based Analysis Plus theoretical framework.

**Results:**

The results of this study are expected in January 2024.

**Conclusions:**

This scoping review endeavors to present a thorough overview of the available evidence concerning how smart technologies interact with older adults over an extended period. The insights gained from this review will lay the groundwork for a research program that explores how older adults adapt to and use smart technologies throughout their lives, ultimately leading to improved user satisfaction and experience and facilitating aging in place with tailored support and user-centered design principles.

**International Registered Report Identifier (IRRID):**

PRR1-10.2196/51129

## Introduction

In recent years, the Internet of Things (IoT) has gained significant attention from researchers, health service providers, entrepreneurs, and technology developers [1,2]. The IoT refers to the interconnection of various devices, objects, and systems through the internet, enabling them to collect and exchange data and perform intelligent actions [1]. The rapid growth of IoT has been fueled by advancements in connectivity, sensors, data analytics, and artificial intelligence [3]. Concurrently, researchers have recognized the potential of various technologies in meeting the needs of a rapidly expanding aging population [2,4,5]. These technologies aim to enhance the quality of life, foster independent living, and address the challenges associated with aging in place.

Older adults (aged 55 years or older) are expected to make up 16% of the global population by 2050 [6]. Ensuring effective support for older adults who choose to “age in place,” meaning they prefer to live independently in their own homes rather than relocating to institutionalized care facilities such as nursing homes, is of utmost importance [7-9].

Smart technologies are believed to have the potential to help older adults successfully age in place [10]. Smart technologies refer to a wide range of devices, systems, and applications that use sensors to collect data that can influence the machines’ “decision-making,” that connect with and share data with other systems, that use processors to run algorithms based on predefined rules or prescriptive models, and that have a user interface of some kind. Lyardet and Aitenbichler [11] define them as having “computational power, information and sensing capabilities into everyday objects and environments.” These systems leverage technology to enhance functionality, automation, and interactivity [12] and are increasingly viewed as tools that expand both capability and capacity to support older adults, many of whom live with multiple chronic conditions and age in their homes [13].

Older adults can benefit from smart technologies to promote independent living [4,14]. Smart technologies can also be customized to a degree to the unique needs and preferences of older adults [15,16] and are increasingly used to support occupational and leisure activities in various aspects of daily life [14,17]. To date, research has demonstrated that smart technologies can promote independent living, enhance health and well-being, and help to improve the quality of life in older adults and their caregivers [14,18-21]. For example, smart wearable devices can serve as fall detection and prevention tools, providing alerts or notifications in case of a fall or sudden changes in movement patterns that can help older adults with their self-management and active engagement [22,23]. Other studies have explored the use of voice-activated virtual assistants like Amazon Alexa or Google Assistant by older adults [24,25]. For example, such assistants can help older adults with their exercise routines, engage in digital leisure, and support self-management of their health and well-being [24-27].

The literature exploring the interaction between smart technologies and older adults, from the young old (65-74 years old) to the oldest old (>85 years old), is limited. However, it is crucial to understand this interaction in order to maximize the benefits these smart technologies can provide. While several studies have explored older adults’ use of smart technologies [14,15,28-30], fewer studies have explored how older adults coadapt to and with these technologies [31-35]. In the context of smart technologies and older adults, coadaptation refers to the process by which both older adults and the technologies they use adapt and adjust to each other over time [36-38]. As an example, a recent longitudinal study of Google Home suggests that patterns of use and benefits experienced by older adults from these technologies change as they get more used to its features and increase their confidence and digital literacy [39].

However, most existing studies fail to consider how older adults coadapt technologies over time to meet their evolving needs. Understanding how older adults adapt to and interact with smart technologies over an extended period is essential for the development of effective and sustainable technological solutions. Moreover, this is important to understand how to sustain adoption as we know from previous research that some abandon it after trial [40]. A recent qualitative systematic review and meta-synthesis explored the existing qualitative data on older adults' perspectives, opinions, and experiences regarding wearable devices [41]. The review synthesized the perspectives of 349 participants, ranging in age from 51 to 94 years, who wore smart devices for durations ranging from 3 days to 24 months [41]. Among other factors, the authors emphasized the importance of the technology’s ability to adapt to user’s preferences over time, as this was found to play a crucial role in determining the long-term adoption of smart technologies by older adults [41].

This scoping review protocol aims to address the existing research gap and to better understand the current body of scientific literature that explores the interaction between smart technologies and older adults over time. Furthermore, the review will explore how their interactions coadapt to optimize user experiences and the benefits they may accrue from their use. This study will support further research on how older adults compensate for declines as they age, how to tailor artificial intelligence–enabled technologies for specific needs, inform the development of more intuitive technologies, alleviate caregiver stress, and develop policies that promote independent living.

## Methods

### Study Design

A scoping review was chosen as the systematic method for synthesizing knowledge [42-44] to comprehensively map the available evidence and provide a broad overview of the literature on the interaction between smart technologies and older adults, considering the diverse range of study designs, methodologies, and sources in this research area [44]. This scoping review protocol was guided by the PRISMA-P (Preferred Reporting Items for Systematic Reviews and Meta-Analyses Extension for Protocols) guidelines [45,46]. Our proposed study will use the methods outlined in the Joanna Briggs Institute manual for knowledge synthesis [47] and will be reported following the PRISMA-ScR (PRISMA Extension for Scoping Reviews) guidelines [48]. Our study will not involve evaluating the quality of the included studies as scoping reviews seek to identify and map all relevant evidence rather than assessing the strength or validity of individual studies [43]. The study will be conducted in 2023.

### Ethical Considerations

Ethics review board is not necessary for this particular study as all literature will be publicly available. The public was not involved in the design of this scoping review protocol. This scoping review protocol has undergone registration in the Open Science Framework [49].

### Theoretical Lens

This study will be informed by 2 theoretical frameworks. This study will be informed by the Selection, Optimization, with Compensation (SOC) framework [50,51] that suggests as individuals age, they use adaptive strategies to maintain or enhance their well-being and achieve their goals, despite changes in cognitive and physical abilities [50,51]. This can be applied to understand how older adults coadapt with smart technology as they age. The selection aspect of the SOC framework refers to the process of choosing goals, activities, and resources that align with one's capabilities and priorities, whereas optimization involves actively engaging in practices to enhance performance and maximize benefits [50,51]. Compensation in the SOC framework refers to the adaptation strategies individuals use to overcome limitations or challenges in achieving their goals, such as using alternative approaches or tools to compensate for declines in cognitive or physical abilities [50,51]. Over the life span, there are 3 main functions associated with ontogenetic development, namely growth, maintenance, and the regulation of loss. The ability to manage weakening functionality and intellectual capacity using SOC lessens as we age, increasing the importance of research into scientific and cultural innovations that will extend the optimization of development and independence later into the lifespan [50].

The study will also incorporate the Sex- and Gender-Based Analysis Plus (SGBA+) theoretical framework [52]. SGBA+ draws on intersectionality frameworks and was specifically chosen to examine sample characteristics, including biological sex and various social positions of older adults, such as ethnicity, income, age, race, education, and gender within the existing body of research [52,53]. This framework was chosen based on the growing recognition that there are significant differences and disparities in older adults’ technology use and adoption experiences related to socioeconomic, demographic, physical, cultural, and psychological factors [54,55].

### Review Questions

What is the extent and nature of the existing scientific literature exploring the coadaptation between smart technologies and older adults and how do older adults and technology coevolve over time to enhance older adults’ experience with the technology?

Subquestions are (1) what have been the outcomes of the coadaption between older adults and smart technology? (2) What specific outcome measures have been used in studies investigating the experiences of older adults in terms of coadapting with technology over time? (3) What specific strategies or approaches have participants used to adapt to the changing circumstances, challenges, or opportunities in their environment, and what process do they follow when making adaptive decisions? (4) What are the key characteristics of older adult participants who have been involved in studies examining the coadaptation between smart technologies and older adults? (5) What methodological strengths, limitations, and recommendations have been documented in the literature regarding the exploration of coadaptation between smart technologies and older adults? (6) What research models and theories contribute to the conceptualization of coadaptation?

### Inclusion Criteria

#### Overview

We used the Population, Concept, and Context (PCC) framework to determine the elements of our inclusion criteria [47].

#### Participants

The participants included in this study will consist of individuals aged 55 years or older and living independently in the community. We operationalize independent living as individuals aging in place, that is, living within houses, apartments, and retirement communities (ie, not institutional care settings [56]). Thus, this review will encompass a diverse population with varying health conditions and levels of independence.

#### Concepts

The concepts of interest are smart technologies, interactions and coadaptations, and experiences and outcomes. Smart technologies, in the context of this study, refer specifically to wearables and voice-activated virtual assistants [57]. Older adults, as end users of smart technologies, gain a progressively refined understanding of the technology's functionalities, operational nuances, and potential utilities [38]. Simultaneously, the technology itself assimilates and accommodates user preferences, habits, and behaviors through machine learning algorithms and adaptive interfaces [36]. In the context of older adults, it is notable that the iterative coadaptation process fosters an environment of familiarity and trust, resulting in reduced cognitive load and an enriched user experience [58,59]. As postulated by Melenhorst et al [60], further research should delve into the intricate interplay of individual differences, cognitive aging, and the evolution of technology in the context of sustained coadaptation over time. Thus, our concepts focus on the dynamic process of interaction between older adults and smart technologies and involve an exploration of how older adults engage with and adapt to the use of these technologies over time. We define coadaptation as the reciprocal adjustments and changes that occur in both older adults and smart technologies as they interact and learn from each other [36,37,61-63]. We will include any study that has explored coadaptation using older adults as the human component of the system. This includes, but is not limited to, quality of life, well-being, social connectedness, independence, and overall user experience.

#### Context

This review aims to encompass a wide range of contexts, settings, disciplines, and fields without imposing limitations. We will include articles from diverse areas such as clinical sciences, public health, social sciences, engineering, and policy. By adopting such a transdisciplinary approach, the review aims to provide a comprehensive understanding of the topic by incorporating insights from various domains and disciplines.

We will limit the search to literature published since 2000, in part to ensure the review can prioritize studies that are more likely to reflect current knowledge, practices, and technologies related to the topic of interest.

### Types of Sources

Our sources will consist of peer-reviewed scientific literature that presents empirical study designs (eg, quantitative research studies, qualitative research studies, mixed methods studies, experimental studies, observational studies, case-control studies, cohort studies, and cross-sectional studies). We will also include conference proceedings and dissertations. By incorporating diverse empirical study designs, the review aims to gather a comprehensive range of evidence and perspectives on the topic, allowing for a more nuanced understanding of the interaction between smart technologies and older adults. Literature reviews and grey literature will be excluded.

### Search Strategy

The search strategy for this scoping review will be developed by an information specialist (Centers for Disease Control and Prevention). The following subject headings and terms related to the following concepts may be included in the search: (1) aged, seniors, older adults, elderly, people with disabilities, cognitive and physical disabilities; (2) wearable electronic devices, voice recognition, artificial intelligence, smart technology, smart assistive technology, virtual reality, ambient intelligence; (3) coadaptation, double-loop learning, coevolution, human-computer interface, human-environment interaction, interaction design, personalization, customization, tinkering, crafting, redesign, modification, sensemaking, hacking, accommodation, mutual adaptation, symbiotic evolution, reciprocal adjustment, interactive iteration, user-technology synergy, dynamic interface refinement, adaptive coevolution, concurrent learning, and bidirectional refinement.

These terms will be combined using Boolean operators (such as AND and OR) to construct search queries tailored to specific databases or search engines.

An initial search will be conducted in Ovid MEDLINE, using text words in article titles and abstracts, as well as index terms, to identify relevant articles. This strategy will undergo press peer review to minimize biases and ensure comprehension prior to being finalized [64]. Once the strategy is finalized in Ovid MEDLINE by the research team, it will be translated and adapted for other databases including OVID Embase, PEDro (Physiotherapy Evidence Database), OVID PsycINFO, EBSCO CINAHL, IEEE Xplore, Web of Science, the Cochrane Library, Scopus, and Global Index Medicus by the information specialist. The aim is to ensure comprehensive coverage of relevant literature by searching multiple databases. The retrieval period for the search will span from January 2000 to August 2023.

The final search will be reported following the PRISMA-S (PRISMA literature search extension)—an extension to the PRISMA Statement for Reporting Literature Searches in Systematic Reviews [65]. To identify any studies that may have been missed during the search process, we will conduct a hand-search of the reference lists of the included articles [44] and conduct forward and backward searching [66]. Additionally, we will consult with experts in the field (eg, authors of the included paper) to gather their insights and recommendations on potentially relevant studies that should be included in the review.

### Study Selection

The search results will be combined in EndNote reference management software and duplicates will be removed [67]. The deduplicated records will be imported into Covidence (Veritas Health Innovation Ltd), a tool for managing and documenting studies during the review process [68]. Screening will occur in 2 phases: level 1 for title and abstract screening and level 2 for full-text screening. To ensure consistency, a pilot test will be conducted on 10 titles and abstracts by all members of the research team to evaluate agreement among reviewers. Any discrepancies and ambiguity regarding the inclusion and exclusion criteria will be resolved through discussion, and another pilot test will be conducted until a consensus is reached. Following the pilot testing, the titles and abstracts will be screened by 2 reviewers against the predetermined inclusion criteria. The authors will use Covidence to vote yes, no, and maybe. Abstracts with uncertainty will undergo full-text review. Potentially relevant sources will be assessed at the full-text stage by the same 2 independent reviewers. Any disagreements in voting between reviewers will be resolved through discussion with the senior responsible investigator at both levels of screening. The reviewers will meet weekly with the research team to discuss the screening process. The following inclusion criteria will be used to screen the literature: (1) be published in the English language in a peer-reviewed journal and include primary data on wearables and voice-activated virtual assistants as the primary smart technology interventions; (2) examine the interaction between smart technologies and older adults over a period of 6 months or more; (3) involve study samples of participants only aged 55 years or older.

### Study Extraction

The research team will create a data extraction form to extract data related to the study aims and questions. Thus, the data extraction process will cover various categories, such as study characteristics (eg, the author’s name, year of publication, country of origin, study purpose or aim, study design, data collection methods, sample size, and study setting), participant characteristics (ie, sex, gender, and other identity constructs), underlying models, theories and frameworks used to inform the research, findings, implications for the coadaptation of technology process, and any quality or limitations identified in the study. We will look for instances of shifts in user behavior patterns over time, reflect on adjustments in interactions with smart technologies, and identify instances where both users and technologies engage in reciprocal feedback loops, thereby leading to iterative adaptations.

Three reviewers (KK, AG, and JM) will first pilot the extraction tool on a subset of the articles. The data extraction tool will be modified and revised as needed during the extraction process for each study included. Any disagreements between the reviewers will be resolved through discussion or by involving additional reviewers if necessary. Next, 1 author (KK) will extract data from the included studies. All relevant data from the studies included will be extracted using free text. By extracting data using free text, the reviewers can capture a wide range of details and nuances, providing a detailed overview of the studies’ content and enabling a thorough analysis of the research findings. The senior responsible investigator will then review all extracted data for accuracy.

### Data Analysis and Presentation

The scoping review will use 3 reporting and presentation strategies to provide an overview of the literature.

First, a basic numerical account will be provided, presenting information on the number of included records, the year of publication, the types of study designs included details of included participants, and the outcome measures, among other relevant factors. This numerical summary will give a quantitative overview of the body of literature.

Next, a directed content analysis [69] will be conducted on the included literature, focusing on the purpose of coadaptation techniques used over time, study findings related to outcomes and experience, and implications for technology design and future research. The content analysis will involve inductive and deductive categories. Deductive categories will be informed by the SOC model to examine how older adults use the 3 strategies of selection, optimization, and compensation [51] in their interactions with smart technologies and the coadaptation process over time. We can examine how these strategies influence their satisfaction, experiences, and outcomes with technology. In addition, the model can help identify any gaps or areas where further support or intervention may be needed to enhance the coadaptation process.

To do so, all authors will read over the extraction to become familiar with the content related to older adults' experiences with smart technologies and coadaptation. The authors will then look for instances where older adults engage in selection, optimization, or compensation strategies in their interactions with smart technologies. This will help us identify key concepts derived from the research objectives, research questions, and the SOC. Once we have identified several initial concepts, we will group them into broader categories that align with the three strategies of the SOC model—selection, optimization, and compensation. This step involves organizing similar categories together based on their conceptual similarity. Through team discussion, these categories will be included in a codebook [70]. One author (KK) will then apply the coding framework to the extracted data and return to the full text of the articles if needed for more context [70]. Once the data have been coded, we will organize the coded content based on the patterns, frequencies, and variations within and across the categories to identify common themes [70]. As we explore themes, we will consider differences in various forms of technology [71]. Through team discussions, we will consider the implications of the findings in relation to the research objectives and research questions.

Finally, participant characteristics will be synthesized by having the first author (KK) map the findings into deductive categories informed by the SGBA+ framework [72], including age, patient population, race and ethnicity, sex and gender, sexual orientation, education, geography disability, language, and technology access and comfort [73].

Thus, the descriptive synthesis will also highlight any study limitations, identify knowledge gaps, and suggest opportunities for future research related to the inclusion of participants and gaps in knowledge.

## Results

The results of this study and the submission of a manuscript for peer review are expected in January 2024.

## Discussion

### Preliminary Findings

The scoping review outlined in this protocol will serve as a foundation for a broader program of research exploring the experiences of older adults and smart technology over time. By expanding upon the proposed work in the scoping review, there is potential to leverage innovative technological solutions and evaluation to support optimized independent aging at home or in other environments such as the workplace, for diverse older adults. Additionally, we anticipate that the results of this scoping review will offer methodological clarity and guidance for exploring the development of coadaption features for technology in this context. The findings from the scoping review will be disseminated through a peer-reviewed scientific journal and conference presentations.

### Limitations

One limitation is that the quality of the studies included will not be assessed. Moreover, we will only include articles that describe empirical evidence and have been published in English over the last 23 years. It is important to acknowledge this limitation as it may bias included studies to those conducted in western countries. Thus, readers of our review must interpret the findings of the review with caution, recognizing that the quality and generalizability of the included studies may vary. The terms coadaptation and smart technologies exhibit significant variation in their usage. During the development of our search strategy, we worked with an information specialist to conduct pilot tests using various terms to ensure comprehensive coverage. However, despite these efforts, there remains some uncertainty as to whether all relevant search terms will be included. As such, it is important to acknowledge that despite our best efforts, which include hand searching and expert consultation, there may still be some risk of missing articles in this rapidly evolving field.

### Conclusions

This scoping review aims to present a comprehensive overview of the existing evidence concerning the interaction between smart technologies and older adults over time. The outcomes of this review will establish the basis for a research program investigating how older adults engage with and adapt to smart technologies across various contexts and throughout their lives. Recognizing the unique requirements of older adults, offering suitable assistance, and integrating approaches that prioritize user needs can substantially enhance the satisfaction and overall experience of older adults who aim to age in their preferred environment with the aid of smart technologies.

## Abbreviations

IoT: Internet of Things
PCC: Population, Concept, and Context
PRISMA-P: Preferred Reporting Items for Systematic Reviews and Meta-Analyses Extension for Protocols
PRISMA-S: Preferred Reporting Items for Systematic Reviews and Meta-Analyses literature search extension
PRISMA-ScR: Preferred Reporting Items for Systematic Reviews and Meta-Analysis Extension for Scoping Reviews
SGBA+: Sex- and Gender-Based Analysis Plus
SOC: Selection, Optimization, with Compensation

## Data Availability

The data sets generated and analyzed during this study are available from the corresponding author upon reasonable request.
